# LINE-1 Transcript Heterogeneity in Non-Small Cell Lung Cancers Is Driven by Host Genomic Context and Conserved Functional Hotspots

**DOI:** 10.3390/cancers18030459

**Published:** 2026-01-30

**Authors:** Yingshan Wang, Kenneth S. Ramos

**Affiliations:** Center for Genomic and Precision Medicine, Texas A&M Institute of Biosciences and Technology, Houston, TX 77030, USA; yingshanwang@tamu.edu

**Keywords:** LINE-1, non-small cell lung cancers, heterogeneity, functional hotspots

## Abstract

**Background**: Long INterspersed Element-1 (LINE-1) retrotransposons comprise 17–20% of the human genome. These retroelements are normally silenced early in embryonic development through epigenetic mechanisms and reawakened during oncogenesis, leading to transcriptional dysregulation, genomic instability, and immune evasion. **Methods**: In the present study, we categorized LINE-1 transcripts across 121 non-small cell lung cancer (NSCLC) cell lines from the Cancer Cell Line Encyclopedia (CCLE) by subfamily, length, orientation, chromosomal origin, and distribution. In addition, high-prevalence insertions were mapped to nearby genes to assess potential functional interactions. **Results**: LINE-1 transcript abundance and length in NSCLC were dominated by evolutionarily young subfamilies, particularly L1HS and L1PA2 through L1PA5. Chromosomal patterns were conserved across NSCLC subtypes, with modest enrichment of L1HS activity on Chromosome 4 and the X Chromosome. The lung squamous cell carcinoma (LSQCC) subtype exhibited the highest total levels of L1HS expression relative to other NSCLC subtypes. Race modestly influenced LINE-1 transcript abundance, with cell lines derived from self-identified African American individuals showing elevated overall LINE-1 and L1HS expression. Age showed a weak positive correlation with total LINE-1 abundance. Integrative analysis revealed recurrent hotspots at 22q12.1 and 20p11.21 that were transcriptionally active across subtypes and coincided with previously reported intact LINE-1 elements active in epithelial cancers. Recurrent insertions were located near cancer-associated genes, including *RB1*, *NEDD4*, *FTO*, *LAMA2*, *NOD1*, and *KCNB2*, implicating LINE-1 activity in cis-regulatory remodeling of oncogenic pathways. **Conclusions**: Together, these findings indicate that LINE-1 transcript heterogeneity in NSCLC is shaped by host genomic architecture and conserved functional hotspots, providing new insights into the mechanisms of genetic and epigenetic dysregulation associated with LINE-1 retroelements.

## Simple Summary

Long interspersed element-1 sequences are normally silenced in healthy cells but frequently reactivated in cancer, where they contribute to genomic instability and transcriptional dysregulation. However, the determinants of their transcriptional heterogeneity in non-small cell lung cancer remain poorly defined. In this study, we systematically characterized locus-specific LINE-1 transcript expression across a large panel of non-small cell lung cancer cell lines to determine how genomic context shapes retrotransposon activity. We show that LINE-1 transcription is dominated by evolutionarily young elements and follows conserved chromosomal patterns across tumor subtypes, with the highest activity observed in lung squamous cell carcinoma. Importantly, LINE-1 expression is anchored by a small number of conserved genomic hotspot loci that remain active across tumor backgrounds and are located near cancer-relevant genes. These findings provide a genome-scale framework for understanding retrotransposon regulation in cancer and highlight conserved LINE-1 loci as potential markers of genome instability and regulatory vulnerability.

## 1. Introduction

Nearly half of the human genome consists of repetitive genetic elements, which are silenced ontogenically via epigenetic mechanisms to preserve genomic integrity [1,2]. Among these, Long INterspersed Element-1 (LINE-1) retrotransposons constitute roughly 17–20% of the human genome [1]. While most LINE-1 copies are truncated or mutated and no longer capable of mobilization, a small subset of 100+ evolutionarily young, full-length elements remains active in the human genome [1,3]. Intact LINE-1 retroelements encode two proteins, ORF1p, an RNA-binding protein, and ORF2p, a reverse transcriptase and endonuclease, that together mediate the ability of LINE-1 to copy and insert itself into new genomic loci through retrotransposition [4]. Active LINE1s are a source of endogenous mutagenesis, and their reactivation in somatic cells is associated with diverse genomic alterations, including aberrant splicing, exon skipping, gene fusions, and chromosomal rearrangements, all of which can disrupt gene expression and promote genomic instability [4,5,6]. In cancer, LINE1 retroelements contribute to tumorigenesis through both retrotransposition-dependent and independent mechanisms [6,7,8,9,10,11]. The former involves insertional mutagenesis, while the latter encompasses the regulatory functions of LINE-1—derived RNAs and proteins. While the oncogenic activity of LINE-1 encoded proteins has been recognized, little is known about the oncogenic activity of the multiplicity of LINE-1 RNAs, including miRNAs, small interfering RNAs (siRNAs), lncRNAs, and LINE-1 chimeric transcripts. These species have been linked with LINE-1 mediated transcriptional interference, modulation of transcriptional genetic networks, interference with host gene expression, and oncogenic reprogramming [8,9,12]. Intact LINE-1 loci can exhibit markedly different activities depending on the local chromatin microenvironment, DNA methylation status, and adjacent regulatory features [13,14,15]. Prior studies have identified certain “hot” LINE-1 loci that demonstrate exceptionally high transcriptional activity and/or retrotransposition across cancers, suggesting that LINE-1 reactivation is not random but instead shaped by the genomic and epigenetic context of individual loci [3].

The characterization of LINE-1 transcriptional heterogeneity in cancer remains technically challenging due to the high sequence similarity of LINE-1 copies, which complicates locus-level quantification using short-read sequencing. Recent advances, such as L1EM, have enabled the dissection of LINE-1 transcript variants, distinguishing among: (1) “LINE-1 only” transcripts derived from autonomous promoters; (2) “3′ run-on” transcripts extending beyond the LINE-1 sequence; (3) “passive sense” transcripts driven by upstream host promoters; and (4) “passive antisense” transcripts initiated from neighboring genes on the opposite strand. These classifications enable the dissection of host-dependent versus autonomous LINE-1 activity, providing a framework for studying how LINE-1 transcription is influenced by its genomic context rather than solely by intrinsic retrotransposon properties [16].

Non-small cell lung cancers (NSCLC) are the predominant form of lung cancers, comprising several major histological subtypes that include adenocarcinoma (LUAD), squamous cell carcinoma (LSQCC), adenosquamous, and giant and large-cell carcinoma. Among these, LUAD is the most prevalent, followed by LSQCC which together constitute 65–70% of all NSCLC [17,18]. Prior studies have shown that elevated LINE-1 ORF1p expression and LINE-1 hypomethylation are common in NSCLC and correlate with tumor aggressiveness and poor prognosis [19,20,21,22]. Despite their distinct molecular and pathological features, both LUAD and LSQCC exhibit LINE-1 hypomethylation associated with higher histological grade and disease progression [23]. Notably, most studies have reported significantly lower LINE-1 methylation levels in LSQCC compared to LUAD, suggesting greater epigenetic deregulation in the squamous subtype [19,24,25,26]. However, the extent, pattern, and genomic determinants of LINE-1 transcriptional heterogeneity among NSCLC subtypes remain poorly understood. Furthermore, whether LINE-1 activation arises from subtype-specific drivers or conserved functional “hotspot” loci shared across cancers has not been systematically explored.

In the present study, we report the findings of a comprehensive analysis of LINE-1 transcript abundance across 121 NSCLC cell lines from the Cancer Cell Line Encyclopedia (CCLE). We identified both subtype-specific and conserved patterns of LINE-1 transcript profiles in cancer cell lines and found that NSCLC profiles are dominated by the younger L1HS and L1PA2–L1PA5 subfamilies, structured by host genomic context, and anchored by recurrent functional hotspots, notably 22q12.1 and 20p11.21. Together, these findings suggest that LINE-1 transcript abundance in NSCLC is not stochastic but instead reflects selective genomic permissiveness, with implications for understanding retrotransposon-mediated genetic regulatory networks and their usefulness to elucidate molecular mechanisms of epigenetic dysregulation and genomic instability associated with LINE-1 retroelements.

## 2. Materials and Methods

### 2.1. Source Data

RNA-sequencing (RNA-seq) BAM files from 121 NSCLC cell lines were obtained from the Cancer Cell Line Encyclopedia (CCLE) [27]. Detailed demographic information and mutation profiles for these cell lines were retrieved from Cellosaurus [28]. The original non-strand-specific RNA-Seq was performed on Illumina HiSeq 2000 or HiSeq 2500 instruments (Illumina, Inc., San Diego, CA, USA) using a large-scale, automated Illumina TruSeq RNA Sample Preparation protocol, with sequence coverage of no less than 100 million paired 101-nucleotide-long reads per sample [27,29].

### 2.2. Quantifying LINE-1 Locus-Specific Expression Using L1EM

LINE-1 RNA was quantified from available RNA-seq data using L1EM (as implemented in the Cancer Genomics Cloud [16,30]). L1EM employs an expectation–maximization algorithm to estimate locus-specific LINE-1 expression, distinguishing proper LINE-1 transcription from passive co-transcription that overlaps LINE-1 sequences but does not support retrotransposition. To quantify intact L1 RNA, a locus with at least two read pairs per million (FPM) assigned was considered a full-length LINE-1 locus [31]. Total intact LINE-1 RNA expression was calculated by summing the FPM values of all intact LINE-1 loci within each cell line.

### 2.3. Identification of L1HS Elements

L1HS loci annotated as “Category 1” by L1EM, indicating a sufficiently intact 5′ end capable of functioning as a promoter, were selected for downstream analysis. These loci represent putatively active or transcriptionally competent L1HS elements. Transductions linked to source elements lacking intact ORF1 and/or ORF2 domains in the reference genome were excluded from analysis.

### 2.4. Annotation of Genes near LINE-1 Insertion Loci

The genomic positions of recurrent L1HS insertion loci were examined using the UCSC Genome Browser (https://genome.ucsc.edu/). Genes located within or proximal to LINE-1 insertion sites were identified and annotated for potential functional relevance.

### 2.5. Statistical Analysis

Statistical analyses were performed using GraphPad Prism (Version 10.6.1 (799)). Statistical significance was determined using an unpaired two-tailed *t*-test, with a significance threshold of *p* < 0.05.

### 2.6. Data Availability

L1EM is publicly available at https://github.com/FenyoLab/L1EM (latest access date: 26 January 2026). It can also be accessed directly on the CGC through the public app portal: https://cgc.sbgenomics.com/public/apps/whm240/l1em-commit/l1em-cptac3-workflow (latest access date: 26 January 2026).

## 3. Results 

### 3.1. Younger LINE-1 Subfamilies Dominate the Transcriptional Landscape of NSCLC Cell Lines

**Figure 1:**
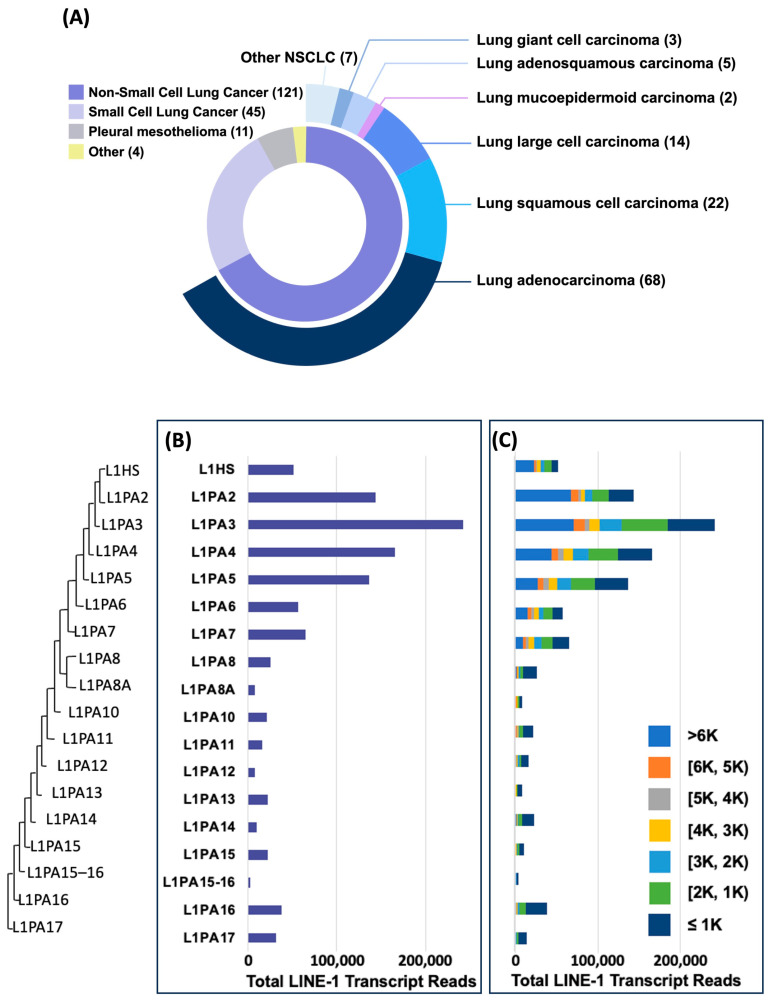
Length and Family Distribution of LINE-1 Transcripts Identified in 121 NSCLC Cell Lines. (**A**) Distribution of 121 non-small cell lung cancer (NSCLC) cell lines from the Cancer Cell Line Encyclopedia (CCLE) database, categorized by pathological subtype. The pie chart shows the percentage of each subtype, with the number of cell lines indicated in parentheses. (**B**) The family distribution of LINE-1 transcript reads from 121 NSCLC cell lines. Phylogenetic tree depicting the evolutionary relationships among various human LINE-1 subfamilies. The bar chart displays the total transcript expression reads for each corresponding LINE-1 subfamily, ordered by their phylogenetic relationship. (**C**) Length distribution of LINE-1 transcript reads for each subfamily.

We profiled the distribution and characteristics of LINE-1 transcripts across 121 NSCLC cell lines from the Cancer Cell Line Encyclopedia (CCLE) (LUAD n = 68; LSQCC n = 22; adenosquamous n = 5; giant & large-cell n = 17). These lines encompass the major histological subtypes and provide a representative spectrum of NSCLC pathology (Figure 1A). Quantitative phylogenetic analysis revealed that LINE-1 transcripts originate from multiple subfamilies, with a pronounced enrichment in evolutionarily younger elements (L1HS and L1PA2 through L1PA5), which contributed the majority of LINE-1 transcript reads (Figure 1B). L1HS, as the youngest subfamily, contributed fewer total reads than L1PA2–L1PA5 yet exceeded most older families, reflecting active but selective transcriptional regulation. Length profiles demonstrated that younger subfamilies generate more full-length (>6 kb) transcripts (notably L1PA2/3), while older families yielded shorter and less abundant transcripts. Few full-length LINE-1 transcripts were detected among L1PA8 and older subfamilies (Figure 1C). These results indicate that NSCLC cells express a broad spectrum of LINE-1 subfamilies, with transcription biased toward evolutionarily younger families. While most of these transcripts may be truncated, their high expression levels suggest active transcriptional regulation and potential biological roles beyond retrotransposition.

### 3.2. NSCLC Subtypes Share a Conserved Pattern of LINE-1 Transcriptional Activity Across Chromosomes

**Figure 2:**
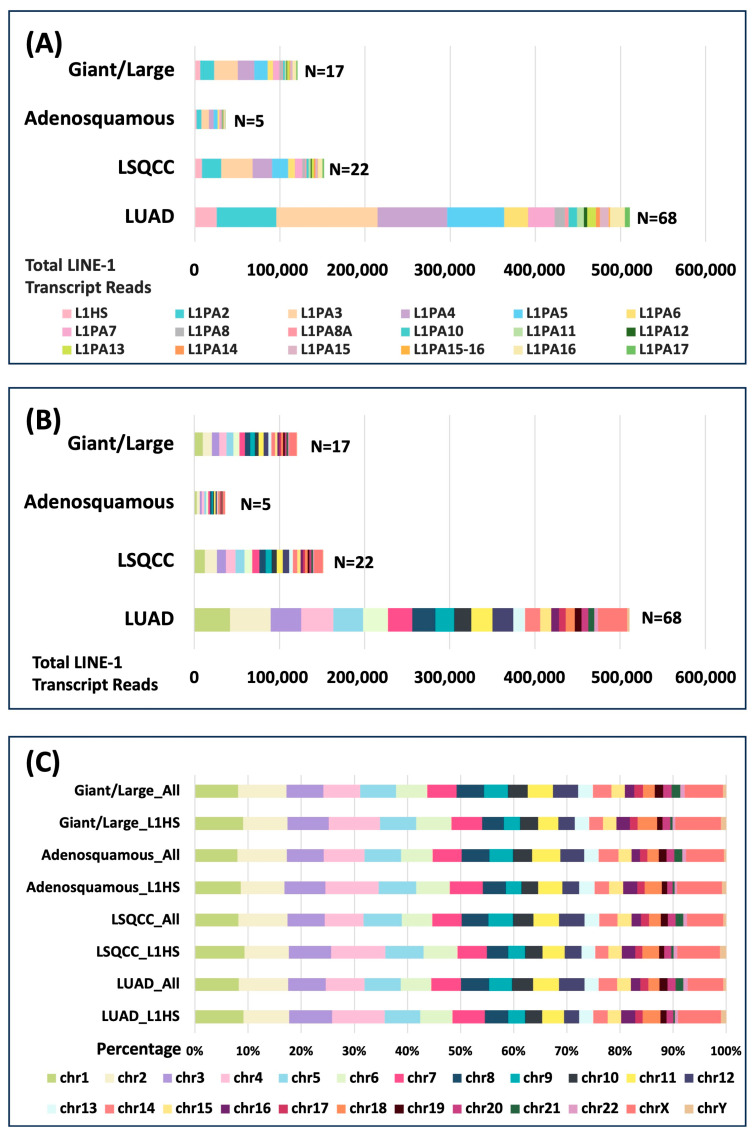
Chromosomal and Subfamily Distribution of Total LINE-1 Transcript Expression in NSCLC Subtypes. (**A**) Subfamily distribution of LINE-1 transcripts stratified by tumor subtype. (**B**) Chromosomal distribution of LINE-1 transcripts stratified by tumor subtype. (**C**) Comparison of chromosomal distribution between all LINE-1 transcripts (All) and L1HS-specific transcripts (L1HS) across tumor subtypes. (Color codification for subfigures (**B**) and (**C**) is the same).

We next examined how LINE-1 transcription varies across NSCLC subtypes and chromosomal locations (Figure 2). Subfamily-level profiling revealed that LINE-1 transcripts in all major NSCLC subtypes share a highly similar subfamily composition, with evolutionarily younger families (L1PA2–L1PA5) contributing the largest proportion of total transcript reads (Figure 2A). Chromosomal distribution of LINE-1 transcripts was non-uniform but positively correlated with chromosome size, with larger chromosomes (chromosomes 1, 2, and X) harboring more LINE-1 reads (Figure 2B). When comparing total LINE-1 transcripts with those specifically derived from the L1HS subfamily, the overall chromosomal patterns were largely consistent across tumor subtypes (Figure 2C). Subtle differences emerged, including increased L1HS transcripts on Chromosomes 4 and the X Chromosome, and relatively higher total LINE-1 representation on Chromosomes 9 and 12. These observations suggest that L1HS expression in cancer cell lines may be concentrated at specific chromosomal hotspots of retrotransposon activity. Overall, no major subtype-specific differences were detected in either subfamily composition or chromosomal distribution, indicating that LINE-1 transcriptional activity is broadly conserved across NSCLC histologies.

### 3.3. L1HS Expression Drives Subtype-Specific Variation in LINE-1 Activity Across NSCLC Cell Lines

**Figure 3:**
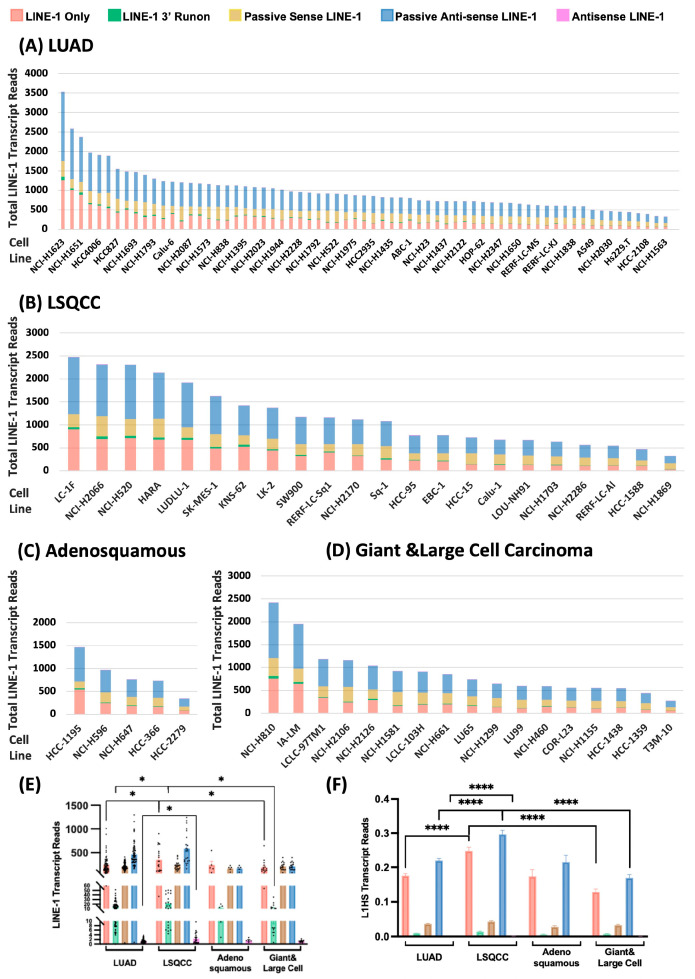
LINE-1 Transcript Profile in NSCLC Cell Lines Stratified by Clinical Classifications. (**A**) Expression of LINE-1 transcripts in lung adenocarcinoma (LUAD) cell lines. The stacked bars represent the total LINE-1 expression level (Reads) for each cell line, and the different colors indicate the contribution of each transcript type: LINE-1 Only, LINE-1 3′ Runon, Passive Sense LINE-1, Passive Antisense LINE-1, and Antisense LINE-1. The cell lines are arranged in descending order of total LINE-1 expression. (**B**) Expression of LINE-1 transcripts in lung squamous cell carcinoma (LSQCC) cell lines, displayed in the same format as Panel (**B**). (**C**) Expression of LINE-1 transcripts in adenosquamous carcinoma cell lines. (**D**) Expression of LINE-1 transcripts in giant & large cell carcinoma cell lines. (**E**) Statistical comparison of total LINE-1 transcript abundance (sum of all intact LINE-1 elements) among subtypes. (**F**) Statistical comparison of L1HS-specific transcript abundance across subtypes (*, *p* ≤ 0.05; ****, *p* ≤ 0.0001; each black dot represents the summed transcript abundance level of all intact LINE-1 elements from one NSCLC cell line).

To further dissect the transcriptional heterogeneity of LINE-1 elements, we stratified LINE-1 transcript expression by pathological subtype (Figure 3). Expression levels varied markedly both across and within subtypes, indicating strong subtype- and cell line (subject)—specific regulation of LINE-1 activity. Across all NSCLC subtypes, passive antisense LINE-1 transcripts driven by non-LINE-1 promoters located within adjacent genomic regions, rather than by autonomous LINE-1 promoter activity, represented the predominant transcript category. This pattern suggests that a substantial portion of LINE-1 expression in NSCLC arises through host-dependent transcriptional regulation rather than intrinsic retrotransposon activation. Within LUAD cell lines, NCI-H1623 exhibited the highest overall LINE-1 expression, while DV-90 displayed the lowest (Figure 3A). Interestingly, NCI-H1623 harbors a TP53 Arg273Leu (c.818G>T) mutation, a well-characterized variant also present in NCI-H2009 and NCI-H1734—two additional LUAD lines ranking among the top five for total LINE-1 transcript abundance. This recurrence is consistent with previous findings showing that TP53 dysfunction contributes to elevated LINE-1 expression. When comparing across histological subtypes, LSQCC cell lines demonstrated the highest overall LINE-1 activity, followed by LUAD, while adenosquamous and giant & large-cell carcinoma lines exhibited markedly lower levels (Figure 3B–D). Statistical comparisons confirmed significant differences in both total and L1HS-specific transcript abundance among subtypes (Figure 3E,F). Notably, the magnitude of variation was greater for L1HS than for total LINE-1 expression, indicating that elevated LINE-1 activity in LSQCC is largely attributable to increased L1HS transcription. Moreover, L1HS enrichment was consistently observed across multiple transcript categories, including LINE-1 only, LINE-1 3′ run-on, and antisense transcripts, further underscoring its dominant contribution to overall LINE-1 activity in this NSCLC subtype. Collectively, these findings reveal pronounced heterogeneity in LINE-1 transcription across NSCLC cell lines and identify L1HS as a key driver of subtype-specific variation, particularly in LSQCC, where both total and autonomous LINE-1 activities are most strongly upregulated.

### 3.4. L1HS Activity Drive Race-Dependent Variation, While Age Modestly Correlates with Total LINE-1 Expression in NSCLC

**Figure 4:**
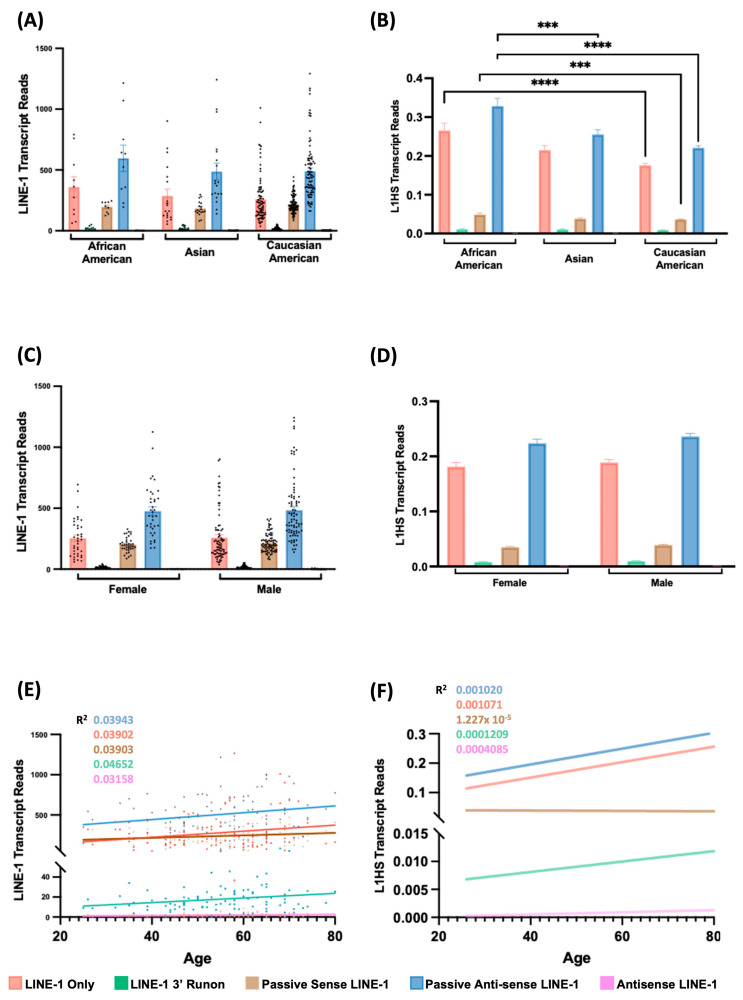
Total LINE-1 Transcript and L1HS Transcript Expression Varies by Tumor Subtype, Race, Sex, and Age. (**A**,**B**) Comparison of total LINE-1 transcript and L1HS transcript expression across different racial groups: African American, Asian, and Caucasian American. (*** *p* ≤ 0.001; **** *p* ≤ 0.0001). (**C**,**D**) Comparison of total LINE-1 transcript and L1HS transcript expression between female and male patients. (**E**,**F**) Scatter plot showing linear regression of each total LINE-1 transcript and L1HS transcript type’s expression against patient age. R^2^ values for each regression line are indicated in matching colors in the top-left corner. (The color key at the bottom denotes the transcript categories: LINE-1 Only, LINE-1 3′ Fusion, Passive Sense LINE-1, Passive Antisense LINE-1, and Antisense LINE-1. Each black or color dot represents the summed transcript abundance level of all intact LINE-1 elements from one NSCLC cell line).

We next examined whether demographic factors contribute to LINE-1 transcriptional variability in NSCLC (Figure 4). Across self-identified racial groups, total LINE-1 transcript levels showed moderate variation, with lines from African American individuals exhibiting slightly higher expression than Asian and Caucasian American samples (Figure 4A). When focusing on the L1HS subfamily, this difference was statistically significant (Figure 4B), suggesting that race-dependent variation in LINE-1 activity primarily arises from differential L1HS expression, rather than global differences across all LINE-1 families. No significant sex-based differences were observed in either total LINE-1 or L1HS transcript abundance between male and female patients (Figure 4C,D), indicating that sex is not a major determinant of LINE-1 transcription in NSCLC. Linear regression analyses revealed weak but positive correlations between LINE-1 expression and patient age (R^2^ < 0.05), suggesting that total LINE-1 activity may modestly increase with age (Figure 4E). However, this trend was weaker for L1HS (Figure 4F), implying that age-associated increases in LINE-1 expression are driven predominantly by older, LINE-1 subfamilies rather than L1HS itself. Together, these observations highlight distinct demographic influences on LINE-1 transcription, with race-related variation primarily reflecting differences in L1HS expression, while age-related trends exhibit broader and modest effects on total LINE-1 activity. Nonetheless, both effects were relatively minor compared with the pronounced subtype-specific differences identified across different NSCLC histologies.

### 3.5. Chromosomal Distribution and Expression of L1HS in NSCLC Cell Lines

**Figure 5:**
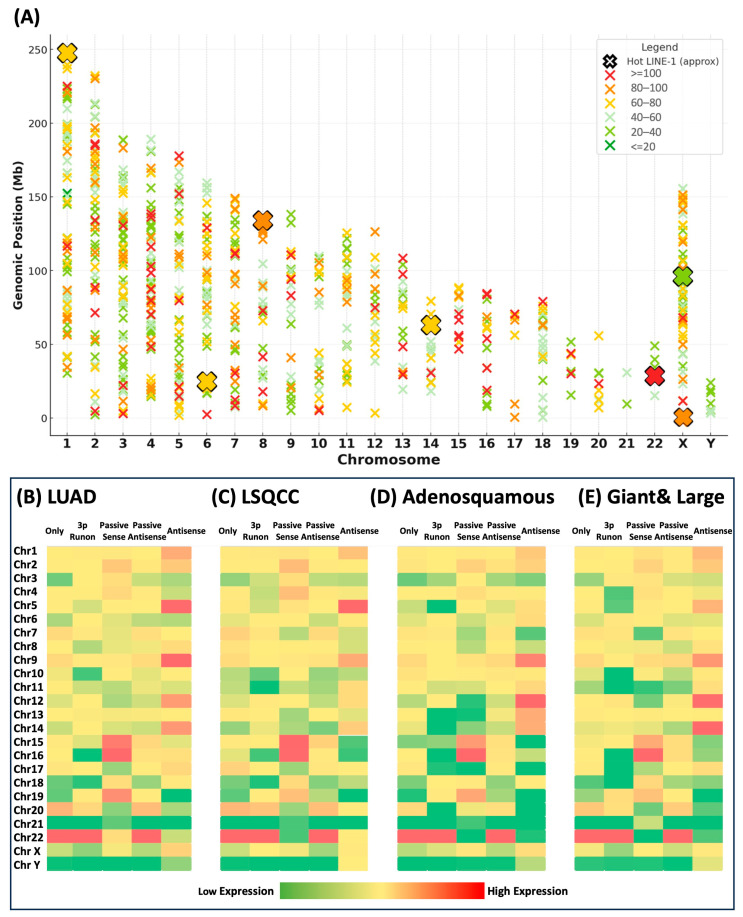
Chromosomal Distribution and Expression of L1HS in NSCLC Cell Lines. (**A**) Genomic distribution of L1HS insertions across chromosomes. Each “x” mark represents an insertion site, colored by insertion frequency. Hot LINE-1 loci are highlighted in red. (**B**–**E**) Average L1HS expression stratified by chromosome and tumor subtype: (**B**) lung adenocarcinoma (LUAD), (**C**) lung squamous cell carcinoma (LSQCC), (**D**) adenosquamous carcinoma, and (**E**) giant & large cell carcinoma. Expression is categorized by transcript type (Only, 3′ Run-on, Passive Sense, Passive Antisense, Antisense). Expression levels are represented on a color scale from low (green) to high (red).

To evaluate the genomic architecture of L1HS activity, we mapped chromosomal insertion and expression patterns across all NSCLC cell lines (Figure 5). L1HS insertions were detected across all chromosomes, with several loci showing high recurrence (>100 of 121 cell lines). Seven well-defined “hot” L1HS loci also exhibit high insertion frequency, suggesting preferred sites of reactivation or retention in NSCLC genomes. Average L1HS expression varied across chromosomes and transcript types, yet the chromosomal distribution pattern remained consistent among tumor subtypes (Figure 5B–E). Chromosome 22 exhibited the highest L1HS expression, largely driven by a single hot LINE-1 locus at 22q12.1. LINE-1 only, 3′ run-on, and passive antisense transcripts displayed highly correlated patterns of chromosomal activity, suggesting coordinated transcriptional regulation. Passive sense transcripts were enriched on Chromosomes 15 and 16, while antisense transcripts showed elevated expression on Chromosomes 5 and 9 in both LUAD and LSQCC, and on Chromosomes 12 and 14 in adenosquamous and giant and large-cell carcinomas. This divergence in transcript-type localization may reflect the influence of local genomic context, such as chromatin accessibility, nearby promoter activity, or the proximity to gene-dense regions, on the directionality and extent of LINE-1 transcription. Despite these subtle distinctions, the overall chromosomal architecture of L1HS expression remained largely conserved across NSCLC subtypes, reinforcing that LINE-1 activation in cancer follows consistent genomic patterns, with selective enrichment at a limited number of recurrent, potentially functional hotspot loci.

### 3.6. Comparison of L1HS Expression and Insertion Patterns Across NSCLC Subtypes

**Figure 6:**
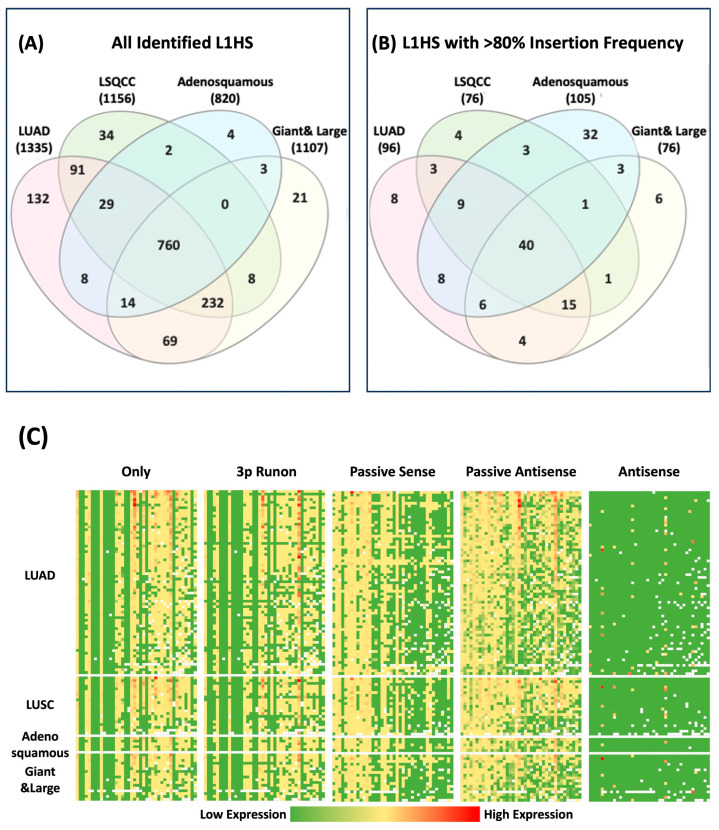
Comparison of L1HS Across NSCLC Tumor Subtypes. (**A**) Venn diagram showing the distribution of L1HS transcripts identified in lung adenocarcinoma (LUAD), lung squamous cell carcinoma (LSQCC), adenosquamous carcinoma, and giant & large cell carcinoma. (**B**) Venn diagram showing L1HS transcripts shared by more than 80% of cell lines within each tumor subtype. (**C**) Heatmap displaying the expression of 40 shared L1HS transcripts across NSCLC cell lines, stratified by tumor subtype and transcript category (Only, 3′ Run-on, Passive Sense, Passive Antisense, Antisense). Each row represents a cell line, and each column represents a LINE-1 insertion locus. Expression levels are shown on a color scale from low (green) to high (red).

To explore the shared and subtype-specific landscape of L1HS activity, we next characterized all the L1HS transcripts identified across NSCLC subtypes, with a focus on those with high insertion prevalence (Figure 6). Venn diagram analysis revealed extensive overlap in L1HS insertions among tumor subtypes, with a large group of 760 L1HS loci shared across all four NSCLC categories, indicating a conserved core of transcriptionally active LINE-1 elements (Figure 6A). Among subtype-specific insertions, LUAD harbored the greatest number of unique L1HS loci, while adenosquamous carcinoma exhibited the fewest, suggesting greater heterogeneity and potential lineage-dependent regulation in LUAD. When focusing on high-prevalence insertions (defined as loci present in >80% of all cell lines), only 40 L1HS transcripts were found to be conserved across subtypes (Figure 6B). This small but consistent subset likely represents a functionally stable group of L1HS loci that remain transcriptionally active in diverse NSCLC backgrounds. Interestingly, within this highly conserved group, adenosquamous carcinoma contributed the most unique L1HS transcripts, whereas LUAD contributed the fewest, indicating that while LUAD has broad L1HS diversity, adenosquamous carcinoma may maintain a smaller yet more stable set of expressed loci.

Expression profiling of these 40 high-prevalence transcripts across all 121 cell lines revealed distinct chromosomal expression patterns, with prominent hotspots at 22q12.1 (corresponding to a previously identified “hot” LINE-1 locus) and at 20p11.21 (Figure 6C). These recurrent regions may represent transcriptionally permissive chromatin environments that sustain consistent L1HS activation across tumor types. Among transcript categories, LINE-1 only and 3′ run-on transcripts displayed the highest degree of expression concordance across cell lines, reflecting similar promoter dependence and processing patterns. These findings indicate that while each NSCLC subtype exhibits unique L1HS signatures, a small subset of loci remains transcriptionally stable and recurrent across varying tumor contexts. These conserved, high-prevalence L1HS loci may represent functionally relevant “core” elements sustaining LINE-1 activity in NSCLC.

### 3.7. Recurrent and Subtype-Specific L1HS Insertions Highlight Potential Oncogenic Pathways in NSCLC

**Figure 7:**
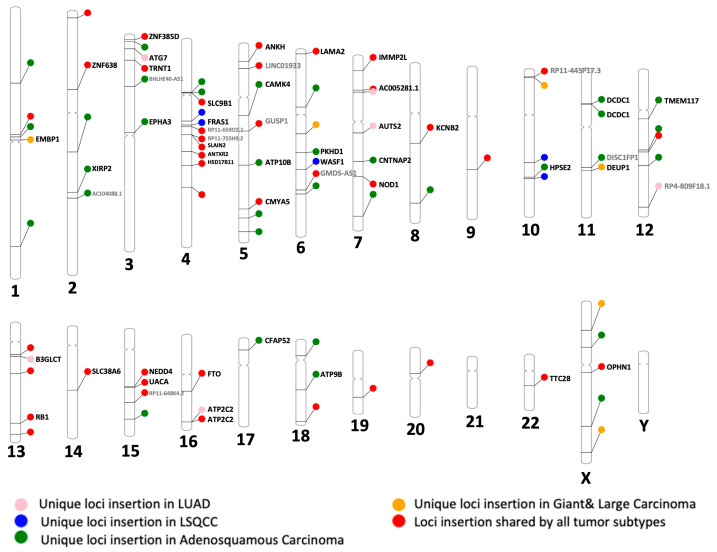
Chromosomal distribution of L1HS insertions shared by ≥80% of NSCLC cell lines, with genes located at the insertion sites.

To further explore the genomic context of high-prevalence LINE-1 activity, we mapped L1HS insertion sites shared by ≥80% of NSCLC cell lines and annotated nearby genes (Figure 7). Several recurrent L1HS insertions observed across all four subtypes were in proximity to well-characterized cancer-associated genes, including *RB1*, *NEDD4*, *FTO*, *LAMA2*, *NOD1*, *KCNB2*, *UACA*, and *ANTXR2*. Many of these genes are dysregulated in NSCLC and play diverse roles in tumorigenesis. These findings suggest that shared L1HS hotspots may mark genomic regions exploited by retrotransposons to influence pathways critical to NSCLC development, while subtype-specific loci highlight distinct evolutionary routes through which LINE-1 insertions reshape signaling networks that promote tumor heterogeneity.

## 4. Discussion

In this study, we provide a comprehensive characterization of LINE-1 transcriptional activity across 121 NSCLC cell lines, representing a diverse spectrum of tumor histologies and genetic backgrounds. Our analyses revealed that LINE-1 transcript heterogeneity in NSCLC arises from the interplay between host genomic context and a limited number of conserved “hotspot” loci that remain transcriptionally active across tumor subtypes. These recurrent regions likely represent functionally permissive chromosomal sites that sustain LINE-1 expression and may contribute to tumorigenesis and genomic instability in NSCLC.

A key confirmatory finding was that the transcriptional landscape of LINE-1 in NSCLC is dominated by evolutionarily younger subfamilies (L1HS and L1PA2–L1PA5), which produce more abundant transcripts than older families. Similar enrichment profiles were described for TCGA cancer types, underscoring their preferential activation across malignancies [32]. This selective enrichment likely reflects targeted vulnerabilities in tumor epigenetic regulation rather than global transposon de-repression [33]. Consistent with prior functional genomic studies, young LINE-1s possess stronger enhancer activity, enabling them to act as distal regulatory elements that modulate nearby gene expression [34]. For instance, a hypomethylated L1PA2 insertion upstream of MET functions as an alternative promoter in several cancers, linking LINE-1 activation to oncogenic signalling [35]. The predominance of full-length L1HS and L1PA2–L1PA3 transcripts in NSCLC suggests that their reactivation may generate functionally competent RNAs capable of modulating chromatin accessibility or generating chimeric transcripts. Together, these observations indicate that preferential activation of younger LINE-1 families is a hallmark of NSCLC and may contribute to transcriptional heterogeneity and tumor progression.

Despite the strong bias toward younger families, the chromosomal distribution of LINE-1 remained remarkably consistent across NSCLC subtypes. Transcript abundance generally correlates with chromosome size, with Chromosomes 1, 2, and X harboring the highest densities of LINE-1 expression. Both total LINE-1 and L1HS transcripts displayed similar chromosomal patterns across subtypes, suggesting conserved genomic rules of activation independent of tumor histology. Subtle enrichments, such as increased L1HS activity on Chromosomes 4 and the X Chromosome, are consistent with prior findings that recent LINE-1 insertions are disproportionately enriched on these two chromosomes [36,37]. LINE-1 elements preferentially integrate into gene-poor regions, AT-rich areas, and the lagging-strand template during DNA replication [38], and our previous full-length synthetic L1RP experiments confirmed a strong bias toward gene-poor regions, such as those found on Chromosome 13 [39]. These findings question the putative random nature of LINE-1 retrotransposition and suggest that the recurrent enrichment of L1HS activity on specific chromosomes arises from inherent genomic and structural constraints. The overall conservation of chromosomal architecture underscores that LINE-1 transcriptional activation in NSCLC is a broadly shared process rather than a subtype-specific event.

At the subtype level, we observed pronounced heterogeneity in LINE-1 transcription, with LSQCC showing the highest total and L1HS transcript levels, followed by LUAD and other histologies. This transcriptional enrichment mirrors our previous finding of elevated ORF1p protein expression in LSQCC compared with LUAD and aligns with reports that LINE-1 activity predominates in epithelial-derived tumors [39]. Notably, multiple studies have demonstrated significantly lower LINE-1 methylation in LSQCC relative to LUAD, reflecting its more extensive epigenetic dysregulation [19,24,25,26,40]. Recent analyses also identified LSQCC as the NSCLC subtype with the largest median gain in LINE-1 expression relative to adjacent-normal tissue (*p* < 1 × 10^−10^) [41]. Among LUAD cell lines, the TP53 Arg273Leu (c.818G>T) mutation was recurrent among high LINE-1–expressing lines (NCI-H1623, NCI-H2009, and NCI-H1734) but absent in other subtypes. Most NSCLC cell lines carry TP53 mutations, and p53 is known to directly bind and repress the LINE-1 5′UTR [42]. In p53-deficient cells, elevated L1HS-derived RNAs correlated strongly with increased ORF1p expression and de novo retrotransposition events [42]. However, de-repression appears stochastic across TP53-mutant contexts, implying that additional regulatory factors determine which loci become active [43]. Our data suggest that specific TP53 mutations, such as Arg273Leu, may differentially influence LINE-1 repression and partially explain the subtype-specific variability in LINE-1 expression observed in NSCLC.

We also identified modest but consistent demographic effects on LINE-1 activity. L1HS expression primarily drives race-dependent variation, with cell lines from African American individuals exhibiting significantly higher L1HS transcript levels than Asian and Caucasian lines. This aligns with prior reports of population-specific differences in LINE-1 methylation and genetic diversity, including elevated polymorphism rates among African Americans [44,45]. Such genetic variation may expand the pool of potentially active elements and increase overall transcriptional diversity. In contrast, sex had no measurable impact, and age-related increases in total LINE-1 expression were modest, likely reflecting mild epigenetic drift rather than robust activation.

Our integrative analysis further revealed that LINE-1 activity in NSCLC is non-randomly organized around recurrent genomic hotspots, with Chromosomes 22q12.1 and 20p11.21 emerging as the most prominent loci. Both regions exhibited high L1HS expression across all subtypes, contributing disproportionately to the overall transcript pool. Notably, the 22q12.1 locus, antisense to an intron of the TTC28 gene, is among the most transcriptionally active and intact LINE-1 elements (full-length and lacking nonsense mutations in ORF1 and ORF2) in breast, ovarian, colorectal, head-and-neck, esophageal, and cervical cancers [13,41,46,47]. The recurrence of this element in NSCLC indicates that 22q12.1 functions as a shared activation hub across multiple epithelial malignancies, consistent with prior TCGA pan-cancer analyses reporting recurrent activation of this locus, particularly in LSQCC [41]. However, most TCGA-based LINE-1 studies have focused on pan-cancer trends or aggregate LINE-1 expression and therefore lack the resolution required to dissect locus-specific transcriptional heterogeneity within lung cancer subtypes. In contrast, our analysis leveraged a large, well-annotated CCLE dataset for systematic characterization of recurrent hotspot loci, chromosomal patterns, and subtype-specific LINE-1 transcriptional programs in NSCLC. Functionally, recurrent L1HS hotspots likely reflect open chromatin and replication timing biases that favor reactivation. Their consistent detection across tumor types and cell lines highlights their potential as biomarkers of LINE-1 activity and genomic instability and as candidate targets for lung cancer therapy.

Finally, mapping of recurrent and subtype-specific L1HS insertions underscores their potential impact on oncogenic signaling and highlights their relevance to clinical and translational oncology. High-prevalence loci were frequently located near well-established cancer genes such as *RB1*, *NEDD4*, *FTO*, *LAMA2*, *NOD1*, *KCNB2*, and *UACA*, implicating LINE-1 insertions in cis-regulatory modulation of cell-cycle control, ubiquitination, RNA modification, and immune signaling pathways. For example, *RB1*, a canonical tumor suppressor, is frequently mutated in NSCLC, with alterations associated with poor prognosis and resistance to immunotherapy [48]; *NEDD4*, overexpressed in up to 80% of NSCLC cases, encodes an E3 ubiquitin ligase that promotes EGFR signaling and metastasis through PTEN degradation [49,50]; *FTO*, whose mRNA and protein are upregulated in NSCLC tissues and cell lines, facilitates tumor growth by increasing USP7 expression and drives LUAD progression via m^6^A demethylation-mediated enhancement of cell migration [51,52]; *LAMA2*, a tumor suppressor, is downregulated in LUAD, where it inhibits metastasis and modulates the *PTEN/PI3K/AKT* signaling axis [53]; *NOD1* functions as an innate immune receptor that mediates inflammatory signaling and may contribute to tumor-promoting inflammation, variants such as rs5743336 have been linked to altered lung cancer susceptibility and treatment response [54]; *KCNB2*, an ion-channel gene, is downregulated in NSCLC, and reduced expression correlates with poor prognosis and diminished immune cell infiltration in LUAD [55,56]; and *UACA*, which regulates apoptosis via APAF1 modulation, is downregulated at both mRNA and protein levels in NSCLC, potentially impairing apoptosome activation and reducing cell death sensitivity [57].

Shared hotspots across subtypes point to conserved mechanisms of LINE-1 reactivation, whereas subtype-specific loci may contribute to lineage-dependent transcriptional diversity. These recurrent insertions thus represent genomic regions susceptible to regulatory rewiring, potentially influencing tumor behavior and therapeutic response. While recent studies have focused largely on LINE-1 ORF1p protein expression as a pan-cancer biomarker, our findings provide novel insights into locus-specific LINE-1 transcripts and reveal distinct activity patterns between lung cancer subtypes [21]. Together, our data support a model in which LINE-1 activation in NSCLC is not random but occurs within permissive chromatin landscapes that intersect with critical cancer-related signaling networks, reinforcing the clinical and translational relevance of locus-resolved LINE-1 profiling.

A limitation of this work is that all inferences were made based on in silico data, and our cell line-based analyses cannot fully capture the complexity of the in vivo tumor microenvironment. Nonetheless, each CCLE cell line represents a distinct patient-derived tumor, providing a controlled and reproducible framework for population-level analyses that would otherwise not be possible using heterogeneous clinical cohorts. In addition, while L1EM enables locus-specific quantification of LINE-1 transcription, it relies on short-read RNA-sequencing data and has the potential for reads from non-reference elements to be misassigned to reference loci. Future studies should experimentally validate the major findings, including the functional relevance of recurrent hotspot loci, the cis-regulatory impact of LINE-1 insertions on nearby oncogenes and tumor suppressors, and the biological consequences of subtype-specific L1HS activation. These will help elucidate the regulatory mechanisms underlying LINE-1 reactivation and define how it interacts with oncogenic signaling networks in NSCLC to drive cancer progression and the acquisition of chemotherapy resistance.

## 5. Conclusions

In summary, this study establishes a comprehensive framework for understanding the transcriptional and genomic architecture of LINE-1 activity in NSCLC. We present evidence that LINE-1 reactivation in lung cancer is dominated by young, enhancer-active subfamilies and structured around conserved genomic hotspots such as 22q12.1. These loci appear functionally integrated into key oncogenic pathways and may serve as biomarkers of lung cancer evolution and genome instability.

## Data Availability

The data generated in this study are available within the article.

## Abbreviations

The following abbreviations are used in this manuscript:

LINE-1	Long INterspersed Element-1
NSCLC	Non-small cell lung cancer
CCLE	Cancer Cell Line Encyclopedia
LUAD	Lung adenocarcinoma
LSQCC	Lung squamous cell carcinoma
L1HS	Human-specific LINE-1 subfamily
